# Cross-cultural adaptation and validation of the Chinese version of the Malocclusion Impact Scale for Early Childhood (MIS-EC/C)

**DOI:** 10.1186/s12955-023-02213-y

**Published:** 2023-12-10

**Authors:** Qiao Chen

**Affiliations:** 1 Department of Stomatology, Women and Children’s Hospital of Chongqing Medical University, Chongqing, China; 2 Department of Stomatology, Chongqing Health Center for Women and Children, Chongqing, China

**Keywords:** Malocclusion, Cross-culturally adaption, Reliability, Validity, Oral health-related quality of life (OHRQoL)

## Abstract

**Background:**

The Malocclusion Impact Scale for Early Childhood (MIS-EC) is a newly developed questionnaire used to measure the parental perceptions of the impact of malocclusion on oral health-related quality of life (OHRQoL) of preschool children aged 3–5 years. This study describes the cross-cultural adaptation and validation of the MIS-EC questionnaire into Chinese version (MIS-EC/C).

**Methods:**

The MIS-EC/C was developed in accordance with international standards. The scale was then evaluated in a cross-sectional study comprising 210 preschool children aged 3–5 years. The reliability of the MIS-EC/C was tested using internal consistency and test-retest reliability analyses. Cross-cultural validity, discriminant validity and convergent validity were tested.

**Results:**

The Cronbach’s α value and intraclass correlation coefficient (ICC) value for the MIS-EC/C were 0.943 and 0.873, respectively. Confirmatory factor analysis indicated that the fitting indicators of the two-factor model all reached the standard. The MIS-EC/C can differentiate preschool children with malocclusion from those without malocclusion. In addition, there is a good relationship between the MIS-EC/C and the general oral health question.

**Conclusion:**

The MIS-EC/C is a reliable and effective assessment tool for assessing the effect of malocclusion on the OHRQoL of preschool children in China.

## Introduction

Malocclusion is a common condition of primary dentition and is caused by the interaction of environmental, genetic, and behavioral factors [1, 2]. It is considered a very common public health problem. Recent study has shown that approximately 56% of children and adolescents worldwide show some type of malocclusion [3]. Some common types of malocclusion in children, such as anterior open bite and posterior crossbite, can affect the aesthetics and oral health-related quality of life (OHRQoL) of children [4–6]. Patient-reported outcomes (PROs) are increasingly being recognized as important in oral epidemiological studies and clinical trials. Subjectively assessing oral health’s impact on mental health, social behavior, and daily functioning is possible with these tools.

In the past few decades, many assessment tools have emerged to evaluate the OHRQoL of children and adolescents [7–9]. However, the number of OHRQoL questionnaires involving preschool children is very small. Currently, there are only three questionnaires designed for this population, i.e., the Early Childhood Oral Health Impact Scale (ECOHIS) [10], the Scale of Oral Health Outcomes for 5-year-old children (SOHO-5) [11], and the Pediatric Oral Health-Related Quality of Life (POQL) [12]. In addition, these three questionnaires mainly address the impact of dental caries on the quality of life. There is no questionnaire that specifically assesses the effects of malocclusion on preschool children. Therefore, it is necessary to develop a questionnaire for preschool children with malocclusion to more sensitively and accurately assess the impact of malocclusion on the quality of life of preschool children. To address this problem, Homem et al. recently developed the Malocclusion Impact Scale for Early Childhood (MIS-EC) [13]. The MIS-EC questionnaire is a tool to assess the parental perceptions of the impact of malocclusion on the quality of life of preschool children aged 3 to 5 years. It includes the impact on the pediatric patients and the family and contains a total of 8 items [13]. The MIS-EC questionnaire was found to be reliable and valid in a preliminary study [13].

The original MIS-EC questionnaire was compiled in Brazilian Portuguese and English. Therefore, to facilitate its use in other languages and cultures, it should be properly translated, followed by cultural adjustments and psychological assessments. To date, no Chinese version of the MIS-EC questionnaire has been verified. Therefore, a Chinese version of the MIS-EC (MIS-EC/C) questionnaire is needed to provide services to Chinese preschool children. In this context, the aim of this study was to translate the MIS-EC questionnaire and adapt it to China’s national context. In addition, the measurement performance of the Chinese version was evaluated to determine whether the MIS-EC/C serves as an effective measure for assessing OHRQoL in Chinese preschool children with malocclusion. The hypothesis is that the MIS-EC/C is similar to the original version and has adequate psychometric properties.

## Methods

### Ethical considerations

This study was approved by the ethics committee of our hospital (no. 2018(009)). Parents of all participants who sought treatment in the Women and children’s hospital of Chongqing Medical University provided written informed consent and comprehended the study’s details.

### Participants

Using convenience sampling, 210 preschool children who sought treatment in the Women and children’s hospital of Chongqing Medical University were recruited. The recommended sample size was 7 times the number of items (8 items*7 = 56), but the confirmatory factor analysis (CFA) required the sample size to be greater than 200 [14]. Thus, the final sample size was 210, which was in compliance with the standard. The exclusion criteria were toothache caused by dental caries, a history of dental trauma one month before the clinical examination and the use of orthodontic appliances in the past.

The questionnaire was applied to all preschool-aged children in the presence of their guardians before the dental examination. If the participants had any questions, the investigators could be contacted at any time. It took approximately 10 min to complete each assessment. The oral examination was performed by an experienced dentist in our hospital (CQ). WHO criteria, i.e., the decayed, missing and filled teeth (dmft) index, was adopted to evaluate caries, which were classified based on number: no caries, dmft = 0; and caries, dmft > 0 [15]. Malocclusion was assessed using previously published standards proposed by Foster et al. [16]. For clinical calibration, repeated examinations were performed on 20 preschool children after one week to confirm the intra-examiner agreement. The Kappa values of the intra-examiner agreement were 0.92 and 0.95, for caries and malocclusion, respectively.

### MIS-EC

The MIS-EC was compiled and studied by Homem et al. [13]. The scale includes 8 items in 2 domains: the Child Impact Sect. (6 items) and the Family Impact Sect. (2 items). For scoring, a Likert scale was used, with scores ranging from 0 (never) to 4 (very often). If the response is “I don’t know,” the item is not scored and considered missing data. The higher the score is, the greater the impact; the total score of the scale ranges from 0 to 32 points. To test the convergent validity, a general oral health question (“How would you evaluate the health of your child’s teeth, mouth, lips and jaws (bones of the oral cavity)?“) was added at the end of the MIS-EC. The possible answers to the general oral health question are “very good (0),“ “good (1),“ “fair (2)” “poor (3)” and “very poor (4).”

### Translation and cross-cultural adaptation

In accordance with standard guidelines [17], I translated and cross-culturally adapted the questionnaire in 5 steps. In the first step, the questionnaire was translated into Chinese by two bilingual translators with dental experience. Two drafts of the questionnaire were then generated. Both translators were proficient in Chinese and English. A second step was to translate the two Chinese versions back into English by two local dental experts who were not familiar with the MIS-EC and resolve the differences between the two versions. Two dental experts and a medical English professor evaluated the translation quality, resulting the second Chinese version. A team of experts studied the conceptual and semantic equivalence of the second draft, revised the draft, and conducted a preassessment with 30 participants. Each item of the evaluation was analyzed separately after the evaluation. We modified the translated version (MIS-EC/C) to keep the original meaning. This was done to avoid any confusion.

### Statistical analysis

Statistical analysis was performed using SPSS software 25.0 and AMOS 25.0 (IBM Corp. NY; USA).A statistical significance level of 0.05 was used.

### Reliability analysis

Internal consistency and test-retest reliability were used to evaluate the reliability of the MIS-EC/C. Internal consistency was evaluated by calculating Cronbach’s α value. Two weeks after completing the MIS-EC/C for the first time, 30 preschool children were randomly selected, and test-retest reliability was evaluated by calculating the intraclass correlation coefficient (ICC). Prior to completing the MIS-EC/C survey, all participants received no treatment.

In general, when the Cronbach’s α value is 0.70 or higher, the difference between the test value and the retest value is comparable. It is recommended, however, the Cronbach’s α value does not exceed 0.95 in order to avoid redundancy [14]. ICC values range from 0 to 1. In general, repeatability is better when the ICC value is high. ICCs can be divided into 4 categories: poor (< 0.50), moderate (0.50–0.75), good (0.75–0.90), and excellent (> 0.90) [18].

### Validity analysis

Cross-cultural validity, discriminant validity, and convergent validity were used to evaluate the validity of the MIS-EC/C. Confirmatory factor analysis (CFA) was used to assess cross-cultural validity. To evaluate the goodness of fit between the model and the data, we used the following parameters: the ratio of chi-square to degrees of freedom (chi-square/DF), the goodness-of-fit index (GFI), the Tucker‒Lewis index (TLI), the comparative fit index (CFI), and root mean square error of approximation (RMSEA). According to traditional standards, an acceptable model has a chi-square/DF lower than 3.0, a TLI greater than 0.90, and a RMSEA less than 0.08 [19].

The discriminant validity of the MIS-EC/C was evaluated using the Mann‒Whitney U test, and the questionnaire scores for participants with and without malocclusion were compared. The convergent validity was evaluated by calculating the correlation coefficient between the MIS-EC/C score and the general oral health question score. Spearman coefficients of 0-0.20, 0.21–0.40, 0.61–0.80, and 0.81-1.0 are broken down into weak, fair, good, and excellent correlations [14]. On the basis of previous study [13], we predicted that there was a good positive correlation between the MIS-EC/C score and the total score of overall oral health problems.

## Results

### Participant characteristics

In this study, 210 preschoolers were included. All preschool guardians said the MIS-EC/C was easy to understand and answered all the questions. No participants answered ‘don’t know’, and no questionnaire item needed to be excluded. It contains the same number of domains and items as the original version. Participants’ basic characteristics are listed in Table 1.The mean age of the participants was 4 ± 1.4 years. There were 92 males (43.8%) and 118 females (56.2%). The majority of preschool children lived in urban areas.In terms of household income, more than half of the households with preschool children had an annual income of more than 50,000 yuan. Regarding the malocclusion status, 64% of the preschool children had malocclusion. In terms of dental caries, the prevalence among the preschool children was 52.9%.

**Table 1:** Characteristics of preschool children (n = 210)

		N (%)
Age (years)	3	21 (10%)
	4	67 (31.9%)
	5	122 (58.1%)
Gender (n)	Male	92 (43.8%)
	Female	118 (56.2%)
Place of residence (n)	Rural	57 (27.1%)
	Urban	153 (72.9%)
Household income (n)	<¥50,000 per year	65 (31.0%)
	>¥50,000 per year	145 (67.0%)
Malocclusion	Absent	107 (51.0%)
	Present	103 (49.0%)
Dental caries	Absent	99 (47.1%)
	Present	111 (52.9%)

### Reliability

Table 2 provides the mean scores and reliability results for the MIS-EC/C.

**Table 2:** Mean scores and reliability results for the MIS-EC/C

Domain	Mean	SD	Corrected item- total correlation	Cronbach’s alpha if item deleted
Child Impact domain				
1. Had difficulty eating or biting certain foods	1.31	0.82	0.803	0.934
2. Had difficulty pronouncing a word	1.28	0.74	0.787	0.935
3. Angry or bad tempered	1.28	0.73	0.787	0.935
4. Acted shy, embarrassed, ashamed or looked worried	1.09	0.88	0.852	0.931
5. Avoided smiling or laughing	0.94	0.70	0.816	0.934
6. Made fun by other children	0.82	0.74	0.772	0.936
Family Impact domain				
7. Felt annoyed or guilty	0.41	0.72	0.805	0.934
8. Had bite problems or teeth positioning problems	0.51	0.87	0.749	0.939

Table 3 provides the internal consistency and test-retest reliability results for the MIS-EC/C. Cronbach’s alpha ranged from 0.935 to 0.959. The total Cronbach’s alpha value for the MIS-EC/C was 0.943. After 2 weeks, 30 preschool children were retested to calculate the reliability (ICC). The ICC values ranged from 0.844 to 0.895. The total ICC score was 0.873.

**Table 3:** Internal consistency and test-retest reliability of the MIS-EC/C

Domain	No. of items	Internal consistency (n = 210)	Test-retest reliability (n = 30)
		Internal consistency (Cronbach’s alpha)	Intraclass correlation coefficient (95% CI)
Total score	8	0.943	0.873 (0.612–0.928)
Child Impact domain	6	0.935	0.844 (0.572–0.917)
Family Impact domain	2	0.959	0.895 (0.673–0.934)

95% CI: 95% confidence interval

### Validity

**Fig. 1:**
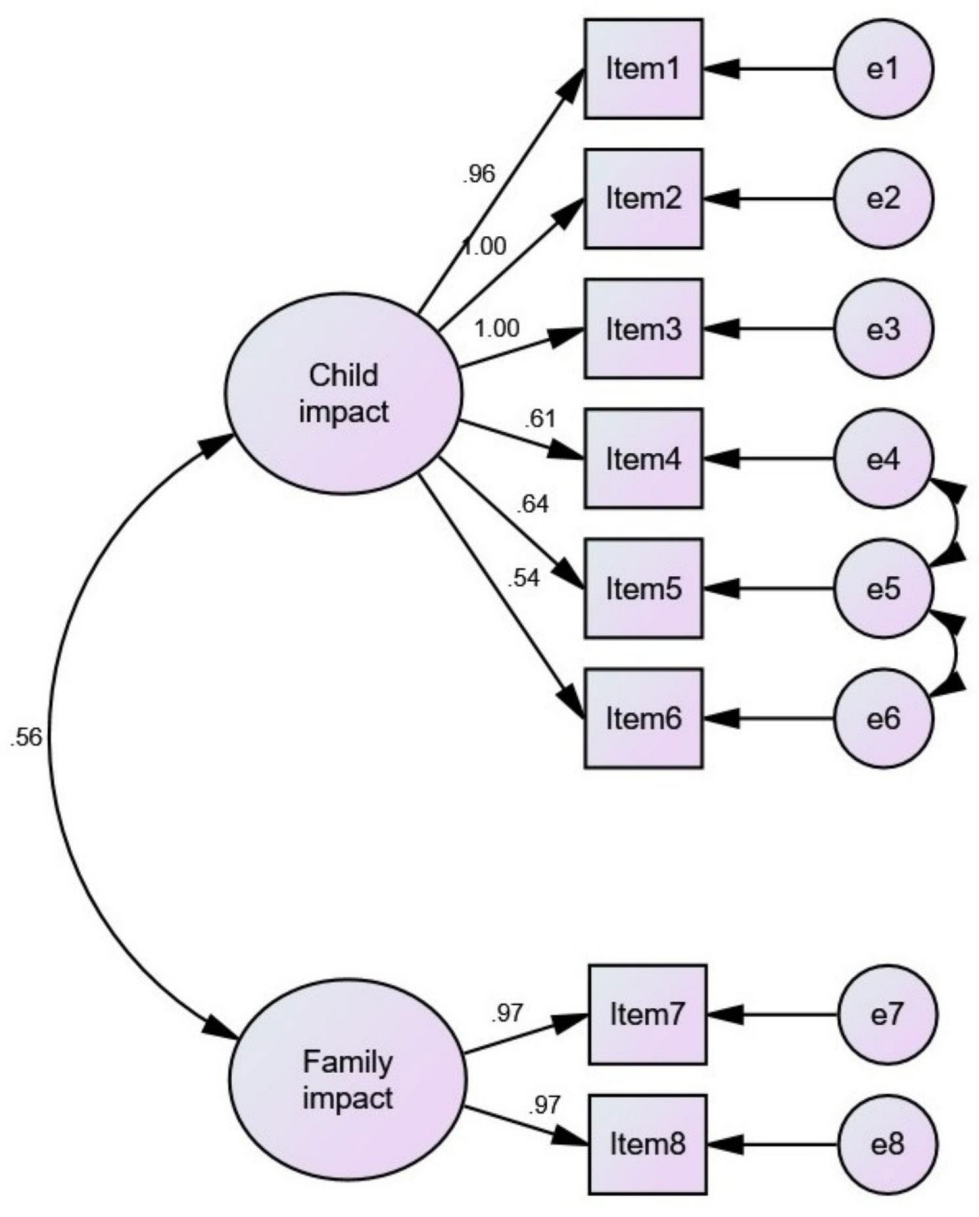
The CFA results of the MIS-EC/C

CFA was used to assess cross-cultural validity. The CFA results indicated that the two-factor model had an acceptable goodness of fit (chi-square/DF = 2.067, GFI = 0.946, TLI = 0.956, CFI = 0.958, and RMSEA = 0.078). The correlation between these factors was high and satisfactory (Fig. 1).

Table 4 provides the discriminant validity results for the MIS-EC/C. Discriminant validity was assessed by comparing the average scores of preschool children with or without malocclusion. The average MIS-EC/C score for the group without malocclusion was 5.59 points and that for the group with malocclusion was 9.76 points. The difference between the two groups was significant, indicating that the MIS-EC/C has good discriminant validity.

**Table 4:** Discriminant validity of the MIS-EC/C

Domain	Malocclusion absent Mean (SD)	Malocclusion present Mean (SD)	*P value*
Total score	5.59 (4.38)	9.76 (5.28)	< 0.001
Child Impact domain	5.00 (3.67)	8.51 (3.59)	< 0.001
Family Impact domain	0.59 (0.91)	1.25 (1.99)	< 0.001

Table 5 shows the convergent validity results for MIS-EC/C. This result indicates that MIS-EC/C has a good relationship with general oral health question.

**Table 5:** Convergent validity of the MIS-EC/C

Domain	*r*_s_*	95% CI	*P value*
Total score	0.598	0.524–0.693	< 0.001
Child Impact domain	0.549	0.426–0.659	< 0.001
Family Impact domain	0.654	0.552–0.752	< 0.001

*Spearman’s rank correlation coefficient

## Discussion

To our knowledge, this is the first cross-cultural translation and adaptation of the English version of the MIS-EC into Chinese, with a subsequent evaluation of its psychometric properties. All guardians of the preschool children reported that they had no difficulty understanding the content of the MIS-EC/C. The results confirmed the hypothesis that the MIS-EC/C had high reliability and validity with regard to measuring OHRQoL in Chinese preschool children.

In recent years, clinical and epidemiological studies on PROs have been conducted by many Chinese researchers, which have been increasingly used to quantify the impact of oral health on diverse populations [20–22]. The cross-cultural adaptation of these scales must strictly follow the procedures proposed by international standards. In China, there are 870 million people who speak Mandarin, so clinical research requires a Chinese version of the MIS-EC questionnaire.

For the reliability analysis, the Cronbach’s α value for the MIS-EC/C was 0.943, higher than the value for the original scale (0.87). The ICC of the test-retest reliability was slightly lower than that for the original scale (0.94), indicating that the MIS-EC/C showed good repeatability when completed at 2 different times. The differences in these results may involve cultural and social factors. Among the relevant literature [23], retest intervals range from 2 days to 1 month. Similar to the assessment of the original scale, we retested participants 2 weeks after the first test. In summary, the results indicate that the MIS-EC/C is a reliable measurement tool.

We performed CFA to evaluate the cross-cultural validity of the MIS-EC/C. Based on the CFA results, it was confirmed that the two-factor model fit the data well. Chi-square/DF and RMSEA are the two most suitable indices for evaluating CFA [24]. It was considered acceptable to have chi-square/DF values of 3.0 and RMSE values of 0.08 in this study.

In addition, questionnaire scores for participants with and without malocclusion were used to evaluate the discriminant validity of the MIS-EC/C. The difference in the average scores for those without malocclusion and those with malocclusion was statistically significant, indicating that the scale had good discriminant validity. According to its convergent validity, the MIS-EC/C shows a good relationship with the general oral health question. These findings are consistent with previous study [13].

The main limitation of this study is that we did not examine the sensitivity of the MIS-EC/C since it requires a longitudinal study. Research will be conducted on the scale’s sensitivity in the future.

In summary, the MIS-EC/C has good reliability and validity and can be applied to preschool children with malocclusion in China for research or clinical purposes. For further validation and improvement of the scale, we need to conduct a sensitivity analysis through a large-scale longitudinal study.

## Conclusions

The MIS-EC/C is a reliable and effective assessment tool for assessing the parental perceptions of the effect of malocclusion on the OHRQoL of preschool children in China.

## Data Availability

The datasets used and/or analyzed during the current study are available from the corresponding author on reasonable request.

## Abbreviations

OHRQoL: The Oral Health-related Quality of Life
MIS-EC: The Malocclusion Impact Scale for Early Childhood
MIS-EC/C: The Chinese version of Malocclusion Impact Scale for Early Childhood
PROs: Patient-Reported Outcomes
CFA: Confirmatory factor analysis
SD: Standard deviation
CFI: The comparative fit index
TLI: The Tucker–Lewis index
SRMR: Standardized root mean square residual
RMSEA: The root means the square error of approximation
ICC: The intraclass correlation coefficient
df: Degree of freedom
CI: Confidence interval

## References

[1] Zou J, Meng M, Law CS, Rao Y, Zhou X (2018). Common dental Diseases in children and malocclusion. Int J Oral Sci.

[2] De Ridder L, Aleksieva A, Willems G, Declerck D, de Cadenas M. Prevalence of Orthodontic malocclusions in Healthy Children and adolescents: a systematic review. Int J Environ Res Public Health. 2022;19. 10.3390/ijerph19127446. 10.3390/ijerph19127446 PMC9223594 35742703

[3] Lombardo G, Vena F, Negri P, Pagano S, Barilotti C, Paglia L, Colombo S, Orso M, Cianetti S (2020). Worldwide prevalence of malocclusion in the different stages of dentition: a systematic review and meta-analysis. Eur J Paediatr Dent.

[4] Kragt L, Dhamo B, Wolvius EB, Ongkosuwito EM (2016). The impact of malocclusions on oral health-related quality of life in children-a systematic review and meta-analysis. Clin Oral Investig.

[5] de Vasconcelos FMT, Vitali FC, Ximenes M, Dias LF, da Silva CP, Borgatto AF, Bolan M, Cardoso M (2021). Impact of primary dentition malocclusion on the oral health-related quality of life in preschoolers. Prog Orthod.

[6] Tondolo Junior J, Knorst JK, Menegazzo GR, Emmanuelli B, Ardenghi TM (2021). Influence of malocclusion on oral health-related quality of life in children: a seven-year cohort study. Dent Press J Orthod.

[7] Alvarez-Azaustre MP, Greco R, Llena C. Oral-health-related quality of life as measured with the Child-OIDP index and oral health status in Spanish adolescents. Int J Environ Res Public Health. 2022;19. 10.3390/ijerph191912450. 10.3390/ijerph191912450 PMC9564813 36231749

[8] Alvarez-Azaustre MP, Greco R, Llena C. Oral health-related quality of life in adolescents as measured with the Child-OIDP questionnaire: a systematic review. Int J Environ Res Public Health. 2021;18. 10.3390/ijerph182412995. 10.3390/ijerph182412995 PMC8701449 34948611

[9] Baherimoghadam T, Hamedani S, Naseri N, Ghafoori A (2022). Validity and reliability of the Persian version of the short-form child perceptions questionnaire 11-14-year-old children (CPQ11-14). Health Qual Life Outcomes.

[10] Pahel BT, Rozier RG, Slade GD (2007). Parental perceptions of children’s oral health: the early childhood oral health impact scale (ECOHIS). Health Qual Life Outcomes.

[11] Tsakos G, Blair YI, Yusuf H, Wright W, Watt RG, Macpherson LM (2012). Developing a new self-reported scale of oral health outcomes for 5-year-old children (SOHO-5). Health Qual Life Outcomes.

[12] Huntington NL, Spetter D, Jones JA, Rich SE, Garcia RI, Spiro A 3. Development and validation of a measure of pediatric oral health-related quality of life: the POQL. J Public Health Dent. 2011;71:185–93. PMC3188947 21972458

[13] Homem MA, Ramos-Jorge ML, Mota-Veloso I, Pereira TS, Martins Junior PA, Normando D, Paiva SM, Pordeus IA, Flores-Mir C, Marques LS (2021). Malocclusion Impact Scale for Early Childhood (MIS-EC): development and validation. Braz Oral Res.

[14] Terwee CB, Bot SD, de Boer MR, van der Windt DA, Knol DL, Dekker J, Bouter LM, de Vet HC (2007). Quality criteria were proposed for measurement properties of health status questionnaires. J Clin Epidemiol.

[15] Davies GN, Barmes DE (1976). An evaluation of proposed revisions to the W.H.O. manual oral health surveys-basic methods. Community Dent Oral Epidemiol.

[16] Foster TD, Hamilton MC (1969). Occlusion in the primary dentition. Study of children at 2 and one-half to 3 years of age. Br Dent J.

[17] Beaton DE, Bombardier C, Guillemin F, Ferraz MB (2000). Guidelines for the process of cross-cultural adaptation of self-report measures. Spine (Phila Pa 1976.

[18] Koo TK, Li MY (2016). A Guideline of selecting and reporting Intraclass correlation coefficients for Reliability Research. J Chiropr Med.

[19] Clark DA, Bowles RP (2018). Model fit and item factor analysis: overfactoring, underfactoring, and a program to Guide Interpretation. Multivar Behav Res.

[20] Wang Y, Li S, Zou X, Xu L, Ni Y (2022). Cross-cultural adaptation and validation of the Chinese version of the loneliness scale for older adults. Geriatr Nurs.

[21] Yang T, Chen W, Lu Q, Sun J (2022). Factor structure and measurement invariance of the Chinese version of the COVID-19 phobia scale in depressive symptoms sample during COVID-19 closure: an exploratory structural equation modeling approach. Front Public Health.

[22] Zhang J, Bai D, Qin L, Song P (2022). The development of the Chinese version of the sports Emotional Intelligence Scale. Front Psychol.

[23] Norquist JM, Girman C, Fehnel S, DeMuro-Mercon C, Santanello N (2012). Choice of recall period for patient-reported outcome (PRO) measures: criteria for consideration. Qual Life Res.

[24] Kang H, Ahn JW (2021). Model setting and interpretation of results in Research using Structural equation modeling: a checklist with guiding questions for reporting. Asian Nurs Res (Korean Soc Nurs Sci).

